# Effects of Different Denaturants on the Properties of a Hot-Pressed Peanut Meal-Based Adhesive

**DOI:** 10.3390/molecules27154878

**Published:** 2022-07-30

**Authors:** Yang Qu, Qin Guo, Tian Li, Hongzhi Liu, Qiang Wang

**Affiliations:** Institute of Food Science and Technology, Chinese Academy of Agricultural Sciences, Key Laboratory of Agro-Products Processing, Ministry of Agriculture, Beijing 100194, China; 13999411257@163.com (Y.Q.); guoqin@caas.cn (Q.G.); 18811777506@163.com (T.L.)

**Keywords:** hot-pressed peanut meal, denaturation, network structure, crosslinking, water resistance

## Abstract

Plant protein-based adhesives could fundamentally solve the problem of formaldehyde-based adhesive releasing formaldehyde, but enhancing bonding strength and water resistance is a necessary measure to realize practical applications. In this study, the effects of different denaturants on the properties of a hot-pressed peanut meal (HPM)-based adhesive before and after crosslinking were studied. Papain, sodium dodecyl sulfate (SDS), urea and crosslinker-polyamide epichlorohydrin (PAE) were used to prepare HPM-based adhesives. The functional groups, bonding strength, thermal behaviors, mass loss, moisture uptake value, viscosity and fracture surface of adhesive samples were analyzed. As a result, (1) papain was used to break HPM protein (HPMP) into polypeptide chains and to reduce the water resistance. (2) SDS and urea unfold the HPMP molecule and expose internal hydrophobic groups to improve the water resistance of the adhesive. (3) A denser network structure was formed by PAE and HPMP molecules, which significantly improved the bonding strength and water resistance of adhesives. In particular, after SDS denaturation and PAE crosslinking, compared with pure HPM adhesive, the wet shear strength increased by 96.4%, the mass loss and moisture uptake value reduced by 41.4% and 69.4%, and viscosity increased by 30.4%. This work provided an essential guide to design and prepare HPM-based adhesives.

## 1. Introduction

Formaldehyde-based adhesives are widely used in the manufacturing of plywood, particleboard and fiberboard, and they are derived from non-renewable fossil sources [1]. In addition, formaldehyde-based adhesive release formaldehyde and free phenol in the process of preparation, transportation and application, which can harm the human body [2]. Therefore, plant proteins [3], lignin [4], starch [5] and other sustainable bio-based raw materials have been used as alternatives to develop new wood adhesives and have a high potential for use in industry. Most studies have focused on the use of different chemical methods to enhance the mechanical performance and water resistance of soybean meal adhesives in recent years [6]. However, soybean meal is mainly used for feed and is affected to the soybean market. Therefore, it is important to make full use of existing protein resources that are considered waste to alleviate protein shortages and to promote sustainable development.

Hot-pressed peanut meal (HPM) is a by-product of pressing peanut oil at high temperature from peanut kernels. [7]. It cannot be used in feed or food processing industry due to the content of aflatoxin exceeding the standard, and thus can only be used as waste [8]. HPM contains more than 45% protein, 87% of which is globulin, which is mainly composed of arachin (glycinin) and conarachin (vicilin) [9]. According to previous reports, HPM protein (HPMP) and soybean protein are similar in their amino acid composition and protein structure; they can both react with compounds to produce adhesives [10]. 

However, HPM adhesive cannot be used in wet environments due to molecular physical entanglement and hydrogen bonding [11]. In order to realize the practical application of HPM adhesives, the structure and properties of natural proteins must be modified to improve their reactivity [12]. Common strategies used in previous reports include denaturation [13], crosslinking [14], and nano-material modification [15]. However, there have been no reports on the effects of different denaturation methods on the properties of HPM adhesive. Therefore, this study can help to develop a more efficient preparation method for HPM-based adhesives.

In this study, different denaturants (papain, SDS, urea) were used to improve the properties of HPM-based adhesives. After the papain treatment, more hydrophilic groups were exposed because HPMP was broken into peptides, which increased the reaction sites and facilitated the cross-linking reaction between HPM and the cross-linking agent [16]. SDS could be inserted into HPMP molecules to break hydrogen bonds and expose hydrophobic groups [17]. The intramolecular hydrogen bonds were destroyed by urea, promoting the unfolding of HPMP molecules [18]. Based on this consideration, three different HPM-based adhesives were prepared through a reaction with PAE. Furthermore, the effects of different denaturation methods on the functional groups, bonding strength, thermal behaviors, mass loss (ML), moisture uptake value (MUV), viscosity and fracture surface of different HPM-based adhesives were studied by means of FTIR, DTG, bonding strength evaluation and viscosity analysis. These results provide a theoretical basis for the further development of HPM-based adhesives.

## 2. Materials and Methods

### 2.1. Materials

HPM (200 mesh, 48.2% protein, 25.8% carbohydrates, 6.39% moisture content, 6.96% ash, 6.87% fiber and 5.14% fat) was obtained from Shandong LuHua grain, oil and Food Co., Ltd. (Shandong, China). SDS, urea and papain were obtained from Shanghai Macklin Biochemical Co., Ltd. (Shanghai, China). Polyamide epichlorohydrin resin (PAE, 25 wt%) was obtained from Zhejiang ChuanHua Huayang Chemical Co., Ltd. (Zhejiang, China). Poplar veneer (25 cm × 25 cm × 0.17 cm, 9.4% moisture content) was obtained from Wenan, Hebei province, China.

### 2.2. Preparation of HPM-Based Adhesive

HPM powder (20 g) and deionized water (55 g) were stirred at 25 °C for 20 min to prepare pure HPM adhesive. Then, 0.8 g of the denaturant agent (papain, SDS and urea) was added into pure HPM adhesive and continuously stirred for 20 min, and the mixtures were marked as HPM-papain, HPM-SDS and HPM-urea adhesive, respectively. Then, PAE (16g) was mixed with different adhesives, and stirred for 10 min at 25 °C, and the mixtures were marked as HPM-papain-PAE, HPM-SDS-PAE and HPM-urea-PAE adhesives, respectively. The formulations are shown in Table 1.

### 2.3. Fourier Transform Infrared (FTIR) Spectroscopy

The samples of adhesive were freeze-dried for 72 h and ground to 200 mesh. Before the test, the sample powder was mixed with KBr at 1:100 and pressed into thin slices. The FTIR spectra were obtained at a wave number from 400 to 4000 cm^−1^ during 64 scans, with 2 cm^−1^ resolution.

### 2.4. Three-Ply Plywood Preparation and Bonding Strength Test

Three layers of plywood were prepared with different adhesive, and the amount of adhesive applied on each layer was 220 g/m^2^. The veneer coated with adhesive was placed in the middle as the core layer, and the grain was perpendicular to the upper and lower sides of the veneer. It was stored at room temperature for 5 min after assembly. Hot pressing was performed at 120 °C, 1.2 MPa and 100 s/mm. The prepared plywood was tested after being left at room temperature for 24 h. A total of 30 specimens (adhesive joint area of 25 mm × 25 mm) were cut from five different plywood samples to determine the dry shear strength (DS) and wet shear strength (WS) according to GB/T 9846-2015 [19]. The DS was tested using a universal testing machine with a crosshead speed of 5 mm/min. For WS, the specimen was immersed in water at 63 °C for 3 h and cooled to room temperature for 10 min, then the WS was measured. 

### 2.5. Thermogravimetry (TGA)

Freeze-dried samples (8 mg dry weight) were weighed in an aluminum crucible. The temperature was increased from 30 °C to 500 °C under a nitrogen flow of 10 mL/min, the heating rate was 20 °C/min [20].

### 2.6. Mass Loss (ML) and Moisture Uptake Value (MUV) Test

ML and MUV measurement were performed according to the scheme of Qu et al. [21]. The adhesive samples were oven-dried at 110 °C to a constant weight (M_a_). The cured adhesive samples were immersed in water at room temperature. After 48 h, the adhesive samples were taken out and dried to a constant weight (M_b_). The formulas were as listed below:

ML (%) = [(M_b_ − M_a_)/M_b_] × 100% (1)

To determine the dry mass, the adhesive samples (8 g) were desiccated to a constant weight at 105 °C. Next, we placed the adhesive samples in a constant-temperature and -humidity incubator at 50 °C and 85% humidity (saturated KCl solution). We recorded the mass of the adhesive sample every 2 h and continued to measure until the sample reached a constant weight. M_1_ and M_2_ were the mass of the samples after MUV and after drying, respectively. The equation was rendered thus:

Moisture uptake (%) = [(M_1_ − M_2_)/M_2_] × 100% (2)

### 2.7. Viscosity Test

The viscosity of adhesive samples was measured at room temperature using a viscometer (DV-III, Ultra, Middleboro, MA, USA), and each sample was measured three times. 

### 2.8. Scanning Electron Microscopy (SEM)

The cured adhesive sample was adhered to the metal table, then the sample was sprayed with gold using JFC-1100E ion sputter (JEOL, Tokyo, Japan). The micromorphology of the adhesive fracture surface was observed using a field-emission scanning electron microscope (Hitachi SU8010).

## 3. Results and Discussion

### 3.1. FTIR Analysis

**Figure 1:**
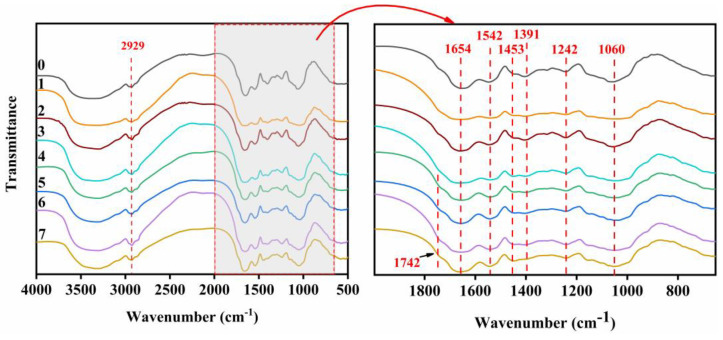
Attenuated total reflection FTIR spectroscopic results from the cured adhesive samples: (0) HPM adhesive, (1) HPM-papain adhesive, (2) HPM-sodium dodecyl sulfate (SDS) adhesive, (3) HPM-urea adhesive, (4) HPM-PAE adhesive, (5) HPM-papain-PAE adhesive, (6) HPM-SDS-PAE adhesive, (7) HPM-urea PAE adhesive.

**Figure 2:**
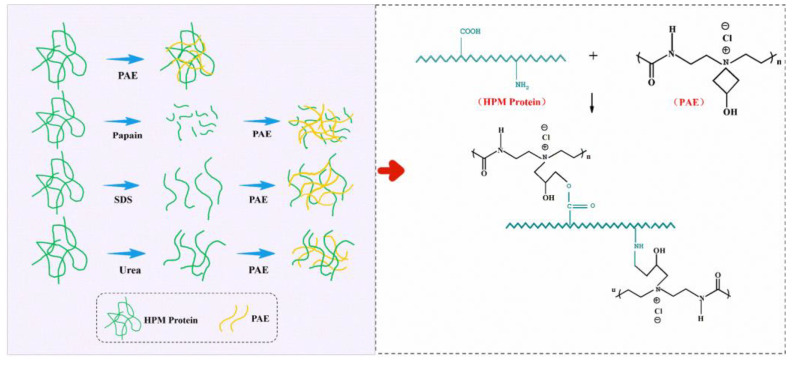
The denaturation and crosslinking mechanism of HPMP molecule.

The structural changes of different adhesive samples were studied by FTIR (Figure 1). The characteristic absorption peaks of HPM were observed at 1654, 1542 and 1342 cm^−1^, which belong to amide C=O stretching (amide I), N-H bending (amide II), C–N stretching and N–H vibrations (amide III), respectively [9]. No new characteristic peaks appeared in the infrared spectrum after adding papain (HPM-Papain adhesive), SDS (HPM-SDS adhesive) and urea (HPM-urea adhesive), demonstrating that the primary structure of the protein molecule had not been changed [22]. Incorporating SDS into the adhesive led to a blue shift of the amide I, amide II, and amide III peaks from 1654, 1542, and 1242 cm^−1^ to 1664, 1545, and 1244 cm^−1^ for the HPM-SDS adhesive, respectively. Similar phenomena were observed in the HPM-urea adhesive, indicating that the intermolecular hydrogen bond of protein was destroyed and more active groups were exposed [16]. With the addition of PAE, a significant blue shift occurred in the characteristic peak of 3369 cm^−1^ (O–H and N–H bending vibrations), indicating that the original hydrogen bond was destroyed and physical bonding was reconstructed [23]. The peak area (COO–) decreased at 1391 cm^−1^, and a new characteristic peak of the carbonyl group appeared at 1742 cm^−1^, which was attributed to esterification of the azacyclobutane group with the carbonyl group of HPMP [21]. In addition, the activation energy of the amino group was lower than that of the carbonyl reaction, which means that it forms a network structure with PAE. The network structure helped to decrease the number of hydrophilic groups in the adhesive and improve the water resistance. The mechanism of the cross-linking is presented in Figure 2.

### 3.2. Bonding Strength Measurement

**Figure 3:**
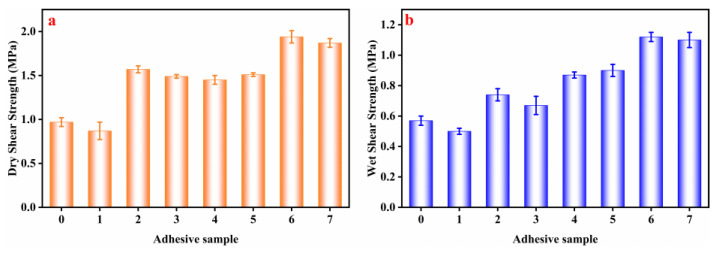
The (**a**) dry shear strength and (**b**) wet shear strength of different adhesive samples: (0) HPM adhesive, (1) HPM-papain adhesive, (2) HPM-sodium dodecyl sulfate (SDS) adhesive, (3) HPM-urea adhesive, (4) HPM-PAE adhesive, (5) HPM-papain-PAE adhesive, (6) HPM-SDS-PAE adhesive, (7) HPM-urea-PAE adhesive.

The DS and WS of the different adhesive samples are presented in Figure 3. The HPM adhesive had a DS of 0.97 MPa and a WS of 0.57 MPa. After adding papain, the HPMP was broken down into polypeptide, the DS and WS of plywood with HPM-papain adhesive were reduced by 10.3% (0.87 MPa) and 40.4% (0.34 MPa) [24]. The ordered structure of native HPMP was denatured as SDS concentration increased, so the DS and WS of HPM-SDS adhesive were significantly increased by 61.9% (1.57 MPa) and 24.6% (0.74 MPa). When the urea was added, the DS (1.49 MPa) and WS (0.67 MPa) of HPM-urea adhesive was also increased significantly. This was due to the secondary structure of the HPMP unfolding, which was conducive to the exposure of hydrophobic groups. The WS of HPM-papain-PAE adhesive, HPM-SDS-PAE adhesive and HPM-urea-PAE adhesive was increased by 57.9% (0.9 MPa), 96.4% (1.12 MPa) and 93% (1.1 MPa) with the addition of PAE, which met the plywood (type II) of Chinese National Standard (GB/T 9846-2015). This was attributed to PAE being distributed into HPM and forming a network structure with the active group (–NH_2_, –COOH) [25]. This result was confirmed by comparison with the HPM-PAE adhesive.

### 3.3. TGA Analysis

**Figure 4:**
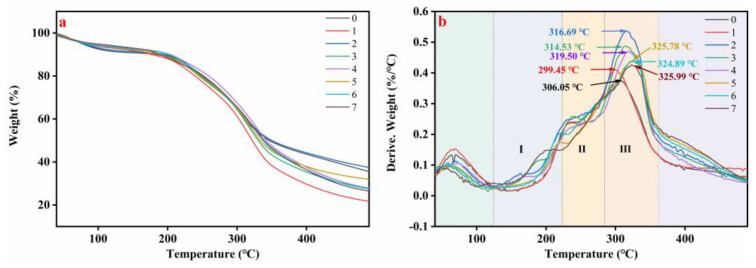
(**a**) Derivative thermogravimetric (DTG) and (**b**) thermogravimetric and curves of the different adhesive samples: (0) HPM adhesive, (1) HPM-papain adhesive, (2) HPM-sodium dodecyl sulfate (SDS) adhesive, (3) HPM-urea adhesive, (4) HPM-PAE adhesive, (5) HPM-papain-PAE adhesive, (6) HPM-SDS-PAE adhesive, (7) HPM-urea-PAE adhesive.

The thermal behaviors of different adhesive samples are shown in Figure 4. The three stages of thermal degradation of the adhesive could be clearly observed (Figure 4a). The first stage occurred over the 123–222 °C temperature range, while the weight loss was attributable to the evaporation of residual moisture [26]. The second stage (222–282 °C) had the most obvious mass loss, which was the decomposition of small molecular substances [27]. Finally, the third stage (282–362 °C) corresponded to adhesive skeleton degradation, including the degradation of peptide bonds and the cleavage of C–N, C–O [28]. After papain treatment, the degradation peak of adhesive 1 moved to a low temperature (299.45 °C), which indicated that the thermal stability decreased. Under SDS and urea, the thermal decomposition temperature of adhesive 2 and adhesive 3 increased by 3.5% (316.69 °C) and 2.8% (314.53 °C), respectively, the improvement could be attributed to the physical enhancement [29]. The thermal degradation peaks of adhesive 5 (324.89 °C), adhesive 6 (325.78 °C) and adhesive 7 (325.99 °C) moved toward higher temperatures with the addition of PAE. Combined with the FTIR analysis, the network structure formed by PAE and HPM could enhance the thermal stability of the adhesive.

### 3.4. ML and MUV Measurement

**Figure 5:**
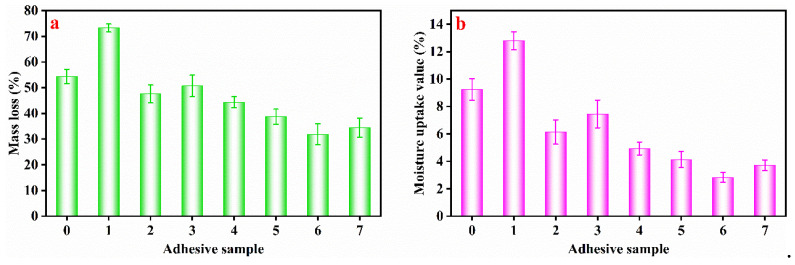
The (**a**) mass loss and (**b**) moisture uptake value of the different adhesive samples: (0) HPM adhesive, (1) HPM-papain adhesive, (2) HPM-sodium dodecyl sulfate (SDS) adhesive, (3) HPM-urea adhesive, (4) HPM-PAE adhesive, (5) HPM-papain-PAE adhesive, (6) HPM-SDS-PAE adhesive, (7) HPM-urea-PAE adhesive.

The ML and MUV were significantly and negatively correlated with the adhesive’s crosslinking degree [30]. Figure 5 showed the ML and MUV of HPM adhesive were 54.37% and 9.25%, respectively. In addition, papain exposed the active hydrophilic groups inside the HPMP molecule, so that the ML and MUV of HPM-papain were increased to 73.25% (Figure 5a) and 12.8% (Figure 5b). As expected, the ML and MUV of HPM-SDS adhesive and HPM-urea adhesive decreased slightly. The ML of HPM-PAE adhesive, HPM-papain-PAE adhesive, HPM-SDS-PAE adhesive, and HPM-urea-PAE adhesive decreased to 44.37%, 38.67%, 31.68% and 34.44%, respectively. The MUV demonstrated the same tendency. This showed that the network structure formed by PAE and HPM significantly improved the water resistance of the adhesive. These results are consistent with the bonding strength analysis.

### 3.5. Viscosity Measurement

**Figure 6:**
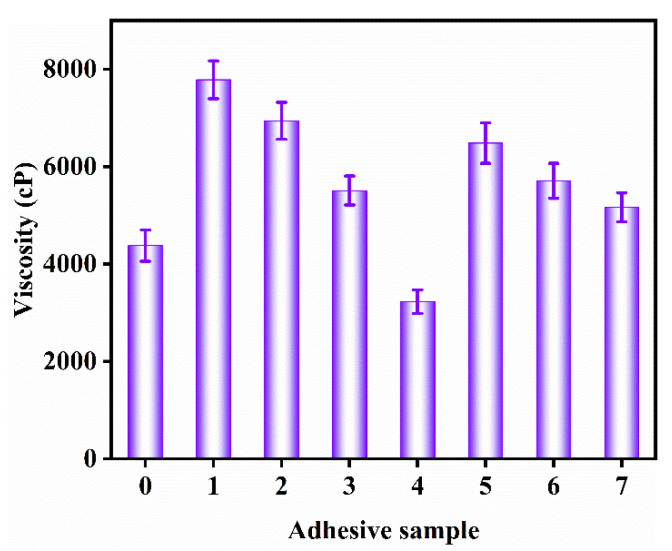
The viscosity of the different adhesive samples: (0) HPM adhesive, (1) HPM-papain adhesive, (2) HPM-sodium dodecyl sulfate (SDS) adhesive, (3) HPM-urea adhesive, (4) HPM-PAE adhesive, (5) HPM-papain-PAE adhesive, (6) HPM-SDS-PAE adhesive, (7) HPM-urea-PAE adhesive.

Viscosity is an important physical property related directly to the flowability of adhesives, and the optimal viscosity of wood adhesives is between 5000 and 25000 mPa·s [24]. HPM adhesive demonstrates over-penetration (Figure 6) in the application process because of its low viscosity (4378 mPa·s) [31]. Therefore, it was unable to form a denser cured adhesive layer during the hot-press cycle, thus having a poor water resistance and bonding strength [32]. The viscosity of the HPM-papain adhesive was increased by 77.8% (7784 mPa·s), and the results indicate that papain hydrolysis broke the peptide bond of HPMP molecules and degraded the molecules into small polypeptide chains [33]. After the introduction of SDS (HPM-SDS adhesive) and urea (HPM-urea adhesive), the viscosity increased by 58.5% (6940 mPa·s) and 25.8% (5509 mPa·s), which was attributed to the reduction in distance and the enhancement of intermolecular friction caused by the destruction of intermolecular hydrogen bonds [9]. Compared with the HPM adhesive, HPM-papain adhesive, HPM-SDS adhesive and HPM-Urea adhesive, the viscosity of the HPM-PAE adhesive, HPM-papain-PAE adhesive, HPM-SDS-PAE adhesive, and HPM-urea-PAE adhesive was decreased by 26.3% (3227 mPa·s), 16.7% (6484 mPa·s), 17.8% (5708 mPa·s) and 6.3% (5162 mPa·s), respectively after adding PAE. There were three reasons for this: (1) the PAE reduced the solid content of adhesives; (2) the positively charged groups of PAE form electrostatic interaction with charged protein chains, resulting in the reduction in attraction and repulsion between surrounding molecules, thus reducing the viscosity [12]; and (3) in the process of crosslinking, PAE could be embedded into molecules and act as “lubricant” [34].

### 3.6. SEM Analysis

**Figure 7:**
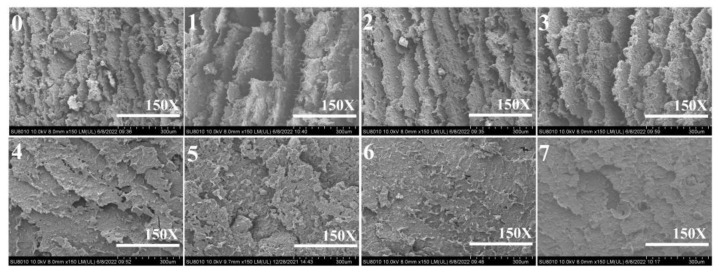
Fracture surface micrographs of the different cured adhesive samples: (0) HPM adhesive, (1) HPM-papain adhesive, (2) HPM-sodium dodecyl sulfate (SDS) adhesive, (3) HPM-urea adhesive, (4) HPM-PAE adhesive, (5) HPM-papain-PAE adhesive, (6) HPM-SDS-PAE adhesive, (7) HPM-urea-PAE adhesive.

Figure 7 shows the fracture surface of the cured adhesive samples. There were a lot of holes and cracks on the surface of the HPM adhesive, which could be used as a channel for water intrusion, resulting in poor water resistance [35]. The above holes and cracks increased after the introduction of papain, indicating that the water resistance of HPMP molecules reduced, degraded by papain. This result was supported by ML and MUV. Although SDS and urea contribute to HPMP molecular unfolding, they would reduce its cohesion, so there were still holes and cracks on the surface of the HPM-SDS adhesive and HPM-urea adhesive. The massive micro-cracks appear on the HPM-PAE adhesive, indicating that the layer was brittle and could easily expose the hydrophobic pathway along the cracks [26]. This was compared with the HPM-papain-PAE adhesive, HPM-SDS-PAE adhesive, and the HPM-urea-PAE adhesive, in which the fracture surface became more compact. The results show that the denaturant treatment was helpful to further improve the crosslinking density of the network, which contributed to the water resistance and thermal behavior of the adhesive.

## 4. Conclusions

In this study, (1) after papain treatment, the WS of the HPM-papain adhesive was reduced by 40.4% (0.34 MPa), the ML and MUV were increased by 34.7% (73.25%) and 38.4% (12.8%), and the viscosity increased by 141.2% (7784 mPa·s). With the addition of PAE, the WS of HPM-papain-PAE adhesive was increased 57.9% (0.9 MPa), the ML and MUV were reduced by 28.9% (38.67%) and 46.6% (4.17%), and the viscosity was decreased by 16.7% (6484 mPa·s). (2) The HPMP molecule hydrogen bond was destroyed under the SDS and urea, the water resistance was improved. Compared with pure HPM adhesive, PAE significantly improved the WS, water resistance and viscosity of the adhesive. The WS of the HPM-SDS-PAE adhesive and HPM-urea-PAE adhesive were increased by 96.4% (1.12 MPa) and 93% (1.1 MPa), the ML was reduced by 41.4% (31.88%) and 36.7% (34.44%), the MUV also showed the same trends (2.83% and 3.71%), and the viscosity was increased by 30.4% (5708 mPa·s) and 17.9% (5162 mPa·s). (3) SDS and urea could improve the water resistance. On this basis, the addition of the PAE improved the bonding strength and water resistance of the prepared plywood, which was due to the cross-linking structure formed during curing and the nail structure formed by the adhesive penetrating into the wood pores.

## Figures and Tables

**Table 1:** Formulations used for adhesive samples.

Adhesive Sample	HPM (g)	Distilled Water (g)	Denaturant (g)	PAE (g)
Pure HPM	20	55	0	0
HPM-Papain	20	55	0.8	0
HPM-SDS	20	55	0.8	0
HPM-Urea	20	55	0.8	0
HPM-PAE	20	55	0	16
HPM-Papain-PAE	20	55	0.8	16
HPM-SDS-PAE	20	55	0.8	16
HPM-Urea-PAE	20	55	0.8	16

## Data Availability

Not applicable.
